# Creep, Hardness, and Elastic Modulus of Lingual Fixed Retainer Adhesives

**DOI:** 10.3390/ma12040646

**Published:** 2019-02-21

**Authors:** Manar N. Hassan, Spiros Zinelis, Monika Hersberger-Zurfluh, Theodore Eliades

**Affiliations:** 1 Clinic of Orthodontics and Pediatric Dentistry, Center of Dental Medicine, University of Zurich, Zurich, Switzerland; Manar.nhassan@gmail.com (M.N.H.); Monika.Hersberger-Zurfluh@zzm.uzh.ch (M.H.-Z.); 2 Department of Biomaterials, School of Dentistry, National and Kapodistrian University of Athens, Athens, Greece; szinelis@dent.uoa

**Keywords:** orthodontic adhesives, mechanical properties, creep, instrumented indentation testing

## Abstract

The aims of this study were twofold: a) to characterize a wide array of time-independent and -dependent properties and b) to find possible correlations among the properties tested. Seven commercially available orthodontic adhesives were included in this study and ten cylindrical specimens were prepared from each material. Five specimens from each material were used for the characterization of Martens Hardness (HM), indentation modulus (E_IT_), and elastic index (η_IT_), and the remaining five for the determination of indentation creep (C_IT_). Al the aforementioned properties were identified by employing an Instrumented Indentations Testing (IIT) device with a Vickers indenter. The results of HM, E_IT_, η_IT_, and C_IT_ were statistically analyzed by one way ANOVA and Tukey post hoc test, while the possible correlations among the aforementioned properties were determined by Spearman correlation test. Statistical significant differences were identified for all properties among the materials tested. Spearman correlation reveals that HM has a positive correlation with E_IT_. Both properties demonstrated a negative correlation with η_IT_ and C_IT_, while no correlation was identified between η_IT_ and C_IT_. Significant differences in the mechanical properties tested may also imply differences in their clinical behavior and efficacy.

## 1. Introduction

Adhesive resins are extensively used in everyday orthodontic practice. The primary application is the cementation of orthodontic brackets on enamel but they also used after orthodontic treatment for fixed retention. Orthodontists use specific orthodontic adhesives for brackets bonding and fix retainers. However common restorative [1,2], flowable composites [3,4], as well as resin composites for universal use [5,6], have been reported for the production of lingual fixed retainers.

Although the mechanical properties of orthodontic adhesives play a significant role on their clinical performance and longevity of bonded devices, it is profound that the requirements for fixed retention are more demanding. Although bonding to dental tissues is a prerequisite for both applications, the demand for high adhesive forces is beneficial for the longevity of fixed retainers as it has been recognized that detachment is the main cause of failure [7]. Two independent studies indicate a survival rate about 60% [5] and 75% [7] of fixed retainers after 4 years of clinical service, which is considered rather low for a device that may be in service for one and sometimes up two decades [8,9]. Therefore, adhesive resins for fixed retainers are more vulnerable to intraoral aging compared to those used for brackets bonding.

Despite the fact that the mechanical properties of adhesives have been tested by experimental and retrieval analysis studies [6,10,11,12,13], there is currently no information on the time-dependent properties of orthodontic adhesives. However, adhesive resins in both aforementioned applications undergo static forces over the whole time of intraoral service. The resin adhesive at the bracket-enamel interface is constantly loaded by the forces applied by orthodontic wires and thus a viscoelastic deformation is anticipated. Although there is no, until today, clinically based evidence, creep may be associated with bracket debonding [14,15]. On the other hand, in fixed retainers, orthodontic adhesives are constantly loaded by the tensile properties exerted by orthodontic wire and thus viscoelastic deformation is also anticipated. However, the viscoelastic properties of orthodontic adhesives have not been thoroughly analyzed and compared among the available materials for lingual fixed retainers [16,17,18].

This study focuses on the characterization of an array of time-dependent and -independent mechanical properties of contemporary orthodontic adhesives, seeking also for possible interrelations among the tested properties. The null hypothesis is that the there are no significant differences in the mechanical properties among the orthodontic adhesives.

## 2. Materials and Methods

### 2.1. Materials

Seven different materials were included in this study. The brand names, the manufactures, the monomer and fillers, as well as the code used in this study, are presented in Table 1. The data were collected from manufactures and previously published papers [11,19,20].

### 2.2. Sample Preparation

Ten cylindrical specimens from each material were prepared. An adequate amount of each material was used to fill the empty space of cylindrical Teflon moulds (h: 3 mm, Ø: 15 mm). The top and bottom of cylindrical spaces were covered with transparent cellulose strips. Then the materials were photopolymerized with slightly overlapping irradiations (40 s each) with a LED curing unit (Radii plus, SDI, Victoria, Australia). The unit emitted light at a standard high irradiance mode (1500 mW/cm^2^). Then the samples were removed from the molds and both surfaces were ground with SiC papers up to 4000 grit employing water as a coolant in a grinding/polishing machine (DAP-V, Struers, Bellarup, Denmark). The purpose of metallographic grinding was twofold; to remove the resin-rich layer and completely level both surfaces of the specimens.

### 2.3. Mechanical Testing

Five specimens for each group were used for the characterization of mechanical properties and the remaining five for the creep index. Both mechanical and creep properties were studied by an Instrumented Indentation Testing (IIT) machine (ZHU2.5/Z2.5 plus test Xpert software, Zwick/Roell, Ulm, Germany). All properties were determined from the directly exposed surfaces to curing unit according to ISO 14557:2002 [21]. Three force-indentation depth curves from each specimen were recorded employing 19.61 N load and 2 s dwell time with a Vickers indenter, and Martens Hardness (HM), indentation modulus (E_IT_), and elastic index (η_IT_) [defined as the elastic to total work ratio], were determined. The mean value of three measurements was used to characterize the specimen itself. The indentation creep (C_IT_) was defined as the percentage increase in indentation depth under constant loading over a given period of time. A constant force 19.61 N was applied for 140 s and the C_IT_ was calculated by the formula

C_IT_ = [(h_2_ − h_1_)/h_1_]*100% (1)

where h_2_ stands for indentation depth at time t_2_ of holding the constant test force and h_1_ the indentation depth at time t_1_ of reaching the test force which is kept constant. Three indentation depth-time curves were obtained from each specimen and the mean value was used to characterize the specimen itself.

### 2.4. Statistical Analysis

The results of HM, E_IT_, η_IT_, and C_IT_ were statistically analyzed by one way ANOVA and Tukey post hoc multiple comparison test employing material type as discriminating variable. Then the possible correlations of C_IT_ with the other three properties were determined by Spearman correlation test. For all cases the level of significance was set at a = 0.05. Statistical analysis was carried out by SigmaPlot (v12) software (Systat Software Inc. San Jose, CA, USA).

## 3. Results

**Figure 1:**
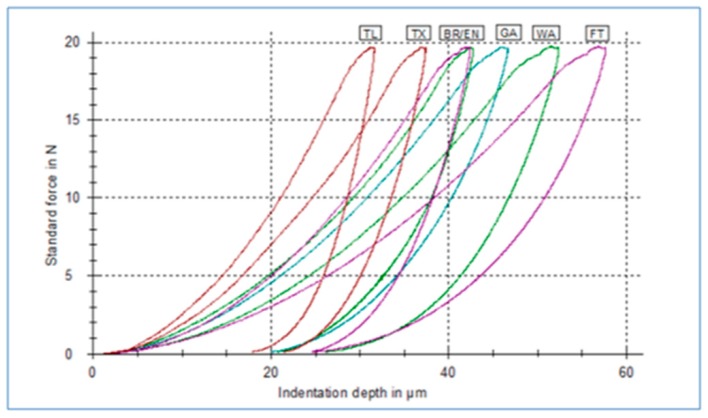
Representative force-indentation depth curves from all materials tested.

Figure 1 demonstrates representative force-indentation depth curves from all materials tested.

The HM increases towards smaller indentation depth at maximum force. A steeper unloading curve after maximum indentation has been reached denotes increased E_IT_ values.

**Figure 2:**
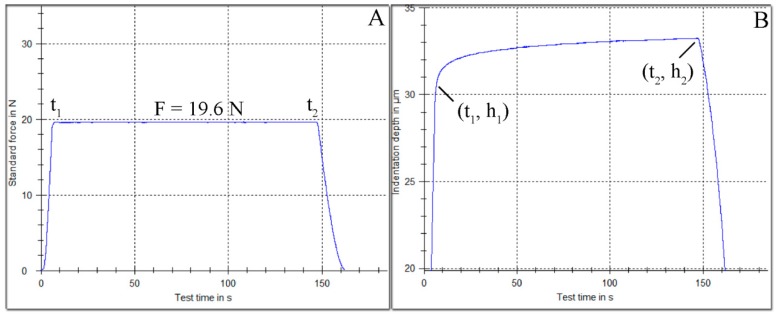
(**A**) A tetragonal pulse where a standard force is applied for a specific period of time. (**B**) The indentation depth of indenter increases over time under constant load.

Figure 2A represents the tetragonal pulse applied while Figure 2B the increase in indentation depth over experimental time.

**Figure 3:**
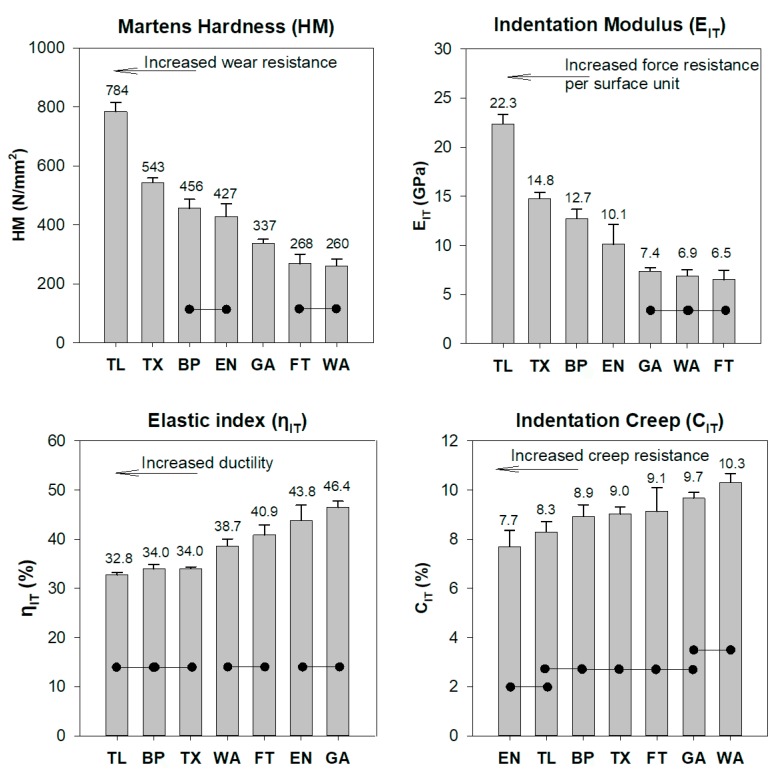
Mean values and standard deviations of all materials tested. Horizontal bars connect materials without statistical significant differences. The properties were sorted in ascending or descending order, starting from the left with the material with the best value for each property.

The numerical results of all mechanical properties along with the statistical outcome for statistical significant differences among materials tested are presented in Figure 3.

**Figure 4:**
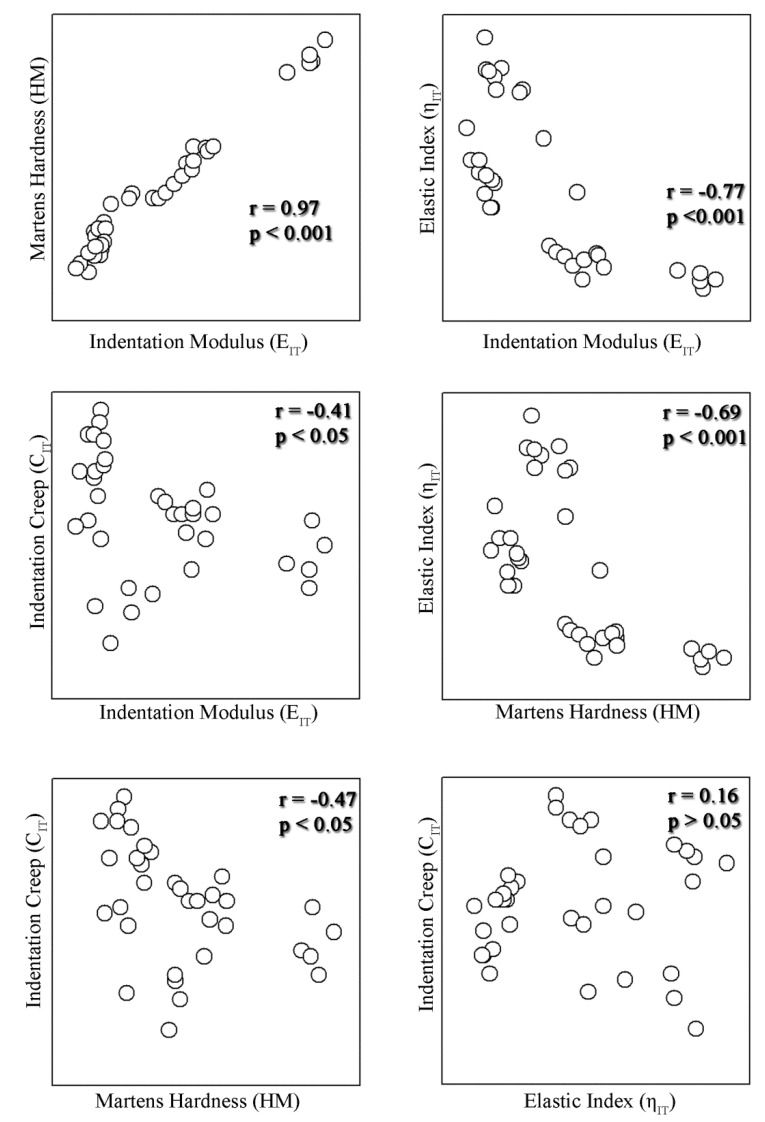
Spearman correlations between the four properties tested. All pairs showed positive or negative correlations apart from the indentation creep with elastic index.

Spearman correlation reveals that HM has a positive correlation with E_IT_. Both properties demonstrated a negative correlation with η_IT_ and C_IT_, while no correlation was identified between η_IT_ and C_IT_ (Figure 4).

## 4. Discussion

According to the results of this study, the null hypothesis must be rejected as significant differences were allocated for all mechanical properties tested among orthodontic adhesives included in this study.

Although HM (also known as universal hardness) is supposedly independent of several limitations of traditional hardness techniques [22], there are limited previously published data of materials tested. Available HM data exists only for products TL (695 N/mm^2^) and TX (568 N/mm^2^) in a previous research [6], both of them close to the experimental findings of this study. The E_IT_ was found in accordance to the data provided by the manufacturer for BP (13.7 GPa) [23]. The results for GA are similar to the data provided by the manufacturer (7.9 GPa) [24] and a previous study after testing in three point bending [19]. The moduli of FT and TL were also found close to previously reported values (FT 5.2 GPa [13]), (TL 17.3 GPa [6,25]) after IIT. The same is true for E_IT_ for TX (15.0 GPa [6]), a value close to one provided by the manufacturer (16.4 GPa [23]) and the value of 14.3 GPa calculated after three point bending [16]. All materials tested showed modulus of elasticity greater than 1.5 GPa, which is the threshold according to ISO 20795-2 for orthodontic base polymers. [26] Elastic index is indicative for the ductility/brittleness of the material tested [27]. A small value indicates a ductile material while a high value indicates a brittle one. The results of this study are close to previously reported values for LR (33.6%) [6,25], TX (30.7%) [6]), and FT (45.0%) [13], while to the best of our knowledge there are no comparable data available on the creep resistance of orthodontic adhesive apart from one recently published study where only one commercially available product was tested [16].

Despite the diversity of monomers and fillers used, all properties tested showed correlations with each other, apart from C_IT_ with η_IT_. The positive correlation between HM and E_IT_ verifies previous findings where Knoop [28], Brinnel [29], and Martens [6] expression of hardness have shown the same trend for resin composites, a finding which is explained by the fact that both properties are positively affected from the increased filler loading. The elastic index demonstrated negative correlation to E_IT_ and HM, denoting that the increase in hardness and modulus is achieved at an expense of ductility. This finding may be attributed to the fact that the increase of both properties is achieved through the increase of filler loading decreasing the capacity of matrix for plastic deformation. On the other hand, indentation creep shows a negative correlation to E_IT_ and HM, as materials with increased filler loading are less susceptible to deformation. The abovementioned correlations declare the complexity to optimize mechanical properties as an array of properties usually increase at the expense of other desirable properties, complicating the formulation of one material for all purposes.

Beyond the correlations among each other, the mechanical properties tested are implicated in the clinical performance of orthodontic adhesives as well. Hardness is the most dominant factor for the wear resistance as lingual retainers are constantly exposed to abrasion due to mastication, and thus materials with increased hardness are preferable for the construction of these devices. It may be noteworthy that lingual fixed retainers may remain in service for even more than a decade [7]. In contrast in the case of brackets bonding, the orthodontic adhesives are not exposed to wear phenomena and thus wear resistance is not a property of importance for this application.

Elastic modulus is basically associated with the geometrical features of lingual fixed retainers, as orthodontic adhesives with higher modulus can provide the same stress resistance with smaller cross sections. Therefore, materials with higher elastic modulus can provide thinner lingual fixed retainers, facilitating patient comfort. An adhesive with higher modulus of elasticity will demonstrate lesser elastic deformation under the constant loading exerted by an activated archwire. Materials with low modulus act as stress absorbers to the system wire-bracket-adhesive tooth tending to store a portion of applied force as elastic energy, as elastic deformation decreases the amount of force transferred to teeth. However, the exact effect of varying the modulus of elasticity on the clinical performance of materials has not yet been quantified and thus the effect of modulus of elasticity has until today only been a theoretical standpoint.

Elastic index is indicative for the ductility of the material [27], and in the case of lingual fixed retainers more ductile materials might be less susceptible to chipping, especially at the adhesive-wire joint where the adhesive resin is much thinner compared with the rest restoration. For brackets bonding, increased creep resistance will decrease deformation over time, mitigating the consequences of a deformed adhesive, as mentioned above for the system wire-bracket-adhesive-tooth. For the lingual fixed retainers, increased creep resistance can preserve more effectively the dimensional stability under intraoral conditions over the whole period of treatment, especially at the adhesive-wire interface where the adhesive is thin.

Although there are differences among materials tested, there is no currently available evidence that time-dependent properties implicated in the clinical efficacy of orthodontic adhesives, and thus this is a promising area for further experimental and clinical research.

## 5. Conclusions

The contemporary orthodontic adhesives demonstrate significant differences in all properties tested.

All mechanical properties tested illustrated correlations with each other, apart from elastic index with indentation creep.

## Figures and Tables

**Table 1:** Commercial names, manufacturers, monomers, filler type, filler loading, and codes of materials tested.

Brand Name (Code) Manufacturer	Monomer	Filler	wt% Inorganic Fillers
BracePaste (**BP**) American Ortodontics Sheboygan, WI, USA	BisEMA TD	BisGMA and BisEMA particles are used as resin fillers Silanated Quartz Silanated Silica	72
Enlight LV (**EN**) Ormco Corporation, Glendora, CA, USA	BisEMA MAOPTMS	Not available (N/A)	N/A
G-aenial Universal Flo (**GA**) GC Corporation, Tokyo, Japan	UDMA, Bis-MEPP, TEGDMA	200-nm Sr glass fillers	69
Flow Tain (**FT**) Reliance Orthodonics Products, Itasca, IL, USA	Bis-GMA TEGDMA	Glass fillers	50
Transbond LR (TR) 3M Unitek Monrovia CA	BisGMA TEGDMA	Silanated quartz	75–85
Transbond XT (**TX**) (for bracket bonding; used for comparison purposes) 3M	Bis-EMA Bis-GMA	Silanated Quartz Silaneted Silica.	70–80
Wave (**WA**) SDI, Victoria, Australia	UDMA BisMEPP TEDMA	Specially treated nano-fillers	65

BisGMA: Bisphenol A-glycidyl dimethacrylated. BisEMA: ethoxylated bisphenol A- dimethacrylate. UEDMA: urethane dimethacrylate. TCDDMA: tricyclodocane. TEGDMA: triethylene glycol dimethacrylate. PCDMA: phenyl-carbamoyloxypropane dimethacrylate. TEDMA: Triethylene glycol dimethacrylate. TEGDMA: triethyleneglycol dimethacrylate. TD: Tetramethylene dimethacrylate. DisMEPP: 2,2-bis[4-(2-methacryloxy)ethoxy)phenyl]propane. MAOPTMS: 3-trimethoxysilylpropyl methacrylate. Bis-MEPP: 2,2-Bis (4-methacryloxypolyethoxyphenyl) propane.
